# P-glycoprotein at the blood-brain barrier: kinetic modeling of ^11^C-desmethylloperamide in mice using a ^18^F-FDG μPET scan to determine the input function

**DOI:** 10.1186/2191-219X-1-12

**Published:** 2011-07-29

**Authors:** Lieselotte Moerman, Dieter De Naeyer, Paul Boon, Filip De Vos

**Affiliations:** 1 Laboratory of Radiopharmacy, Faculty of Pharmaceutical Sciences, Ghent University, Ghent, Belgium; 2 Department of Civil Engineering, Institute Biomedical Technology, Ghent University, Ghent, Belgium; 3 Laboratory for Clinical and Experimental Neurophysiology (LCEN), Department of Neurology, Ghent University Hospital, Ghent, Belgium

## Abstract

**Purpose:**

The objective of this study is the implementation of a kinetic model for ^11^C-desmethylloperamide (^11^C-dLop) and the determination of a typical parameter for P-glycoprotein (P-gp) functionality in mice. Since arterial blood sampling in mice is difficult, an alternative method to obtain the arterial plasma input curve used in the kinetic model is proposed.

**Methods:**

Wild-type (WT) mice (pre-injected with saline or cyclosporine) and P-gp knock-out (KO) mice were injected with 20 MBq of ^11^C-dLop, and a dynamic μPET scan was initiated. Afterwards, 18.5 MBq of ^18^F-FDG was injected, and a static μPET scan was started. An arterial input and brain tissue curve was obtained by delineation of an ROI on the left heart ventricle and the brain, respectively based on the ^18^F-FDG scan.

**Results:**

A comparison between the arterial input curves obtained by the alternative and the blood sampling method showed an acceptable agreement. The one-tissue compartment model gives the best results for the brain. In WT mice, the *K*_1_/*k*_2 _ratio was 0.4 ± 0.1, while in KO mice and cyclosporine-pretreated mice the ratio was much higher (2.0 ± 0.4 and 1.9 ± 0.2, respectively). *K*_1 _can be considered as a pseudo value *K*_1_, representing a combination of passive influx of ^11^C-desmethylloperamide and a rapid washout by P-glycoprotein, while *k*_2 _corresponds to slow passive efflux out of the brain.

**Conclusions:**

An easy to implement kinetic modeling for imaging P-glycoprotein function is presented in mice without arterial blood sampling. The ratio of *K*_1_/*k*_2 _obtained from a one-tissue compartment model can be considered as a good value for P-glycoprotein functionality.

## Background

Multidrug transporters, with P-glycoprotein (P-gp) as most investigated, are a large family of ATP-binding cassette membrane proteins, which appear to have been developed as a mechanism to protect the body from harmful substances [1]. In the blood-brain barrier (BBB), P-gp are responsible for pumping toxic compounds out of the brain, resulting in low concentrations of endogenous and exogenous compounds in the brain. Moreover P-gp overexpression has been observed in brain tissues, obtained after surgery in some epileptic patients [2-4], and could also play a role in other neurological diseases. Since these studies are invasive, it would be useful to have a noninvasive method to predict if P-gp is upregulated in patients.

P-gp function can be studied *in vivo *with radiolabelled substrates. Desmethylloperamide is a metabolite of loperamide, a licensed antidiarrheal agent without central nervous system side effects because P-gp excludes it from the brain [5]. ^11^C-desmethylloperamide (^11^C-dLop) is believed to be the most promising tracer to evaluate P-gp function in the brain [6]. One of the standard methods to investigate the P-gp function in particular, is the use of P-gp knock-out mice. The combination with P-gp blocking studies will give an unquestionable indication of the P-gp function [7].

The objective of this study is the implementation of a kinetic model for ^11^C-dLop and the determination of a typical parameter for P-gp functionality in mice. To set up a kinetic model, it is essential to obtain an arterial input curve, especially if there is no reference region available. Since arterial blood sampling, the gold standard to obtain arterial input curves is very difficult in mice because of the small size and fragility of the mouse blood arteries; an alternative method to acquire the arterial plasma input curve for the kinetic model is proposed.

## Methods

### Animals

Male P-gp knock-out (KO) (Mdr1a (-/-)) mice were purchased from Taconic (Hudson, NY, USA) and male wild-type (WT) mice (FVB) were purchased from Charles River Laboratories (Brussels, Belgium) or Elevage Janvier (Le Genest Saint Isle, France). The study was approved by the Ghent University local ethical committee, and all procedures were performed in accordance with the regulations of the Belgian law. All mice had access to food and water *ad libitum *before the start of the study.

During the entire scan procedure, the animals were kept under anesthesia with 1.5% isoflurane (Medini N.V., Oostkamp, Belgium) administered through a mask and were placed on a heating pad (37°C).

### Radiosynthesis

**Figure 1:**
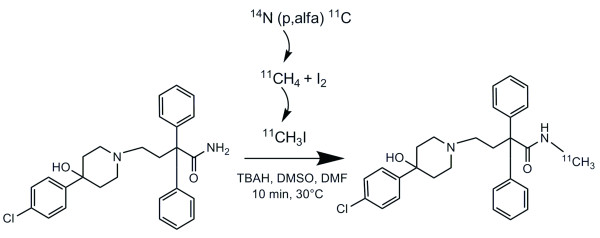
**Radiosynthesis of ^11^C-desmethylloperamide**. Didesmethylloperamide is methylated with ^11^CH_3_I to obtain ^11^C-desmethylloperamide in the presence of tetrabutylammoniumhydroxide, dimethylsulfoxide, and dimethylformamide.

The synthesis of ^11^C-dLop was performed by the methylation of the precursor didesmethylloperamide with ^11^C-iodomethane (Figure 1) as reported earlier by our institution [8]. Didesmethylloperamide was kindly provided by Janssen Pharmaceutica (Beerse, Belgium), while tetrabutylammoniumhydroxide, *N*,*N*-dimethylformamide and dimethylsulfoxide were purchased from Sigma-Aldrich (Bornem, Belgium).

### Comparison of ^11^C-dLop left heart ventricle time-activity curve and blood counter measurement time-activity curve

WT mice (*n *= 3) were anesthetized with isoflurane (1.5%) and cannulated with a polyethylene catheter (60 cm, PE10), filled with heparinised saline (0.9%). One end of the catheter was inserted in the carotid artery of the mice by a precise operation, and at the other end, a syringe needle was inserted. The animals were fixed on the μPET scanner, the catheter was inserted inside the detector and the withdrawing syringe was placed on the main pumping unit as described by Convert *et al. *[9]. Both the μPET scanner (LabPet8; resolution, 1.5 mm) and microvolumetric blood counter (Gamma Medica-Ideas, Quebec, Canada) acquisitions were started in synchronization and subsequent 20-MBq ^11^C-dLop, dissolved in 100 to 150 μl saline/ethanol mixture (9/1, *v*/*v*) was injected intravenously (i.v.). Blood was collected at a constant rate of 10 μl/min for the entire 30-min acquisition time, and the blood time-activity curve was displayed in real time by the software of the microvolumetric blood counter. Immediately after the end of the ^11^C-dLop scan, the mice were injected with 18.5 MBq of ^18^F-FDG in a tail vein. Twenty minutes after ^18^F-FDG injection, a static μPET scan was started for 20 min.

**Figure 2:**
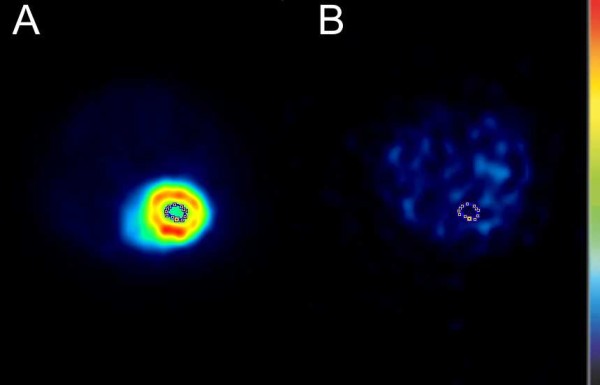
**Transversal image of the mice after injection with ^11^C-desmethylloperamide and ^18^F-FDG**. The ROI delineates the left heart ventricle on the **(A) **^18^F-scan and **(B) **^11^C-scan images (color scale: black, lowest radioactivity uptake; red, highest radioactivity uptake).

Dynamic ^11^C-dLop PET data were sorted into frame sequences of 5 s (*n *= 12), 10 s (*n *= 6), 1 min (*n *= 4), 2 min (*n *= 2), 5 min (*n *= 2), 10 min (*n *= 1). A region of interest (ROI) was drawn manually around the left ventricle of the heart (Figure 2A) on the ^18^F-FDG scan images. Since the position of the mice was unaffected between the ^11^C-dLop and the ^18^F-FDG scan, the ROI of the left heart ventricle on the ^18^F-FDG scan could be pasted on the ^11^C-scan images (Figure 2B) to derive an arterial blood input function. Data from the blood counter were corrected for dispersion with the following formula: *C*_a_(*t*) = *g*(*t*) + *τ*_disp _× (d*g*/d*t*), where *C*_a_(*t*) is the real whole blood activity curve in mice, *g*(*t*) the measured data and d*g*/d*t *the derivative of *g*. *τ*_disp_, the dispersion factor was calculated according to Convert *et al. *[9].

The estimated input function (^18^F-FDG-derived) and the measured input function (blood counter) were compared by a direct and indirect method. The direct method, as described by Fang and Muzic [10], evaluated the input functions by calculating the area under the curve (AUC) difference. Indirect comparison examined the impact of the estimated ^18^F-FDG-derived input function on an estimated kinetic parameter from the kinetic model, like the *K*_1_/*k*_2 _ratio, as described later on (see PET data analysis and kinetic modeling of ^11^C-dLop). The AUC difference was calculated as absolute values of (AUC_PET _- AUC_bloodcounter_)/AUC_bloodcounter _× 100 and the error percentage of *K*_1_/*k*_2 _ratio as absolute values of (*K*_1_/*k*_2PET _- *K*_1_/*k*_2bloodcounter_)/(*K*_1_/*k*_2bloodcounter_) × 100.

### Kinetic model for ^11^C-dLop

#### PET experiments

Before positioning the anesthetized mice on the scanner, WT mice (*n *= 3) were injected i.v. 30 min before the tracer injection with saline (100 μl, controls, *n *= 3) or 50 mg cyclosporine/kilogram body weight (*n *= 3) (Novartis, Vilvoorde, Belgium). Approximately 20 MBq of ^11^C-dLop, dissolved in 100 to 250 μl saline/ethanol mixture (9/1, *v*/*v*) was administered via a tail vein, and the dynamic μPET scan was initiated. After the ^11^C-dLop scan, the mice were injected with approximately 18.5 MBq of ^18^F-FDG in a tail vein (100 μl). Twenty minutes after the ^18^F-FDG injection, a static μPET scan was started for 20 min. KO mice (*n *= 3) were handled in the same way as the WT mice, with exception of the pretreatment procedure.

#### Determination of percent parent compound in plasma and plasma-whole blood ratio of ^11^C-dLop

The determination of percent parent compound (^11^C-dLop) in plasma over time was performed in WT (pretreated with saline or 50 mg cyclosporine/kilogram body weight, *n *= 3 per group and per time point) and KO mice (*n *= 3 per time point) using a high-performance liquid chromatography (HPLC) assay. Thirty minutes after pretreatment, the mice were injected with 22.2 to 30 MBq of ^11^C-dLop (300 μl) and were killed at 1, 10, and 30 min postinjection (p.i.). Blood was collected by cardiac puncture, and the brain was excised. Plasma (200 μl) was obtained after centrifugation (3,000 g, 6 min). Subsequently, 800 μl and 1 ml of acetonitrile (Chem-Lab N.V., Zedelgem, Belgium) were added to the brain and plasma, respectively. Both samples were vortexed (1 min), centrifuged (3,000 g, 3 min), and counted for radioactivity. A supernatant was isolated and analyzed with an HPLC system (Grace Econosphere C18, 10 μm, 10 × 250 mm, eluted with acetonitrile/20 mM sodium acetate (70/30, *v*/*v*) as mobile phase at 7 ml/min). Elution fractions of 30 s were collected and counted for radioactivity. Percent parent compound was calculated as the sum of the counts determined in the fractions containing ^11^C-desmethylloperamide (determined by co-injection with cold desmethylloperamide and UV detection at 220 nm) divided by the total counts of all collected fractions.

To determine the plasma-whole blood ratio, the mice (*n *= 3) were injected with 4.80 to 5.55 MBq of ^11^C-dLop (300 μl) and were killed at 0.5, 1, 2, 3, 5, and 10 min p.i.. Blood was collected from the heart by cardiac puncture, counted for radioactivity, and centrifuged for 10 min (3,000 g). Plasma and blood pellet were separated, weighted, and counted for radioactivity. To obtain the plasma-to-whole blood ratio, counts from plasma and blood pellet were averaged for weight.

#### PET data analysis and kinetic modeling of ^11^C-dLop

**Figure 3:**
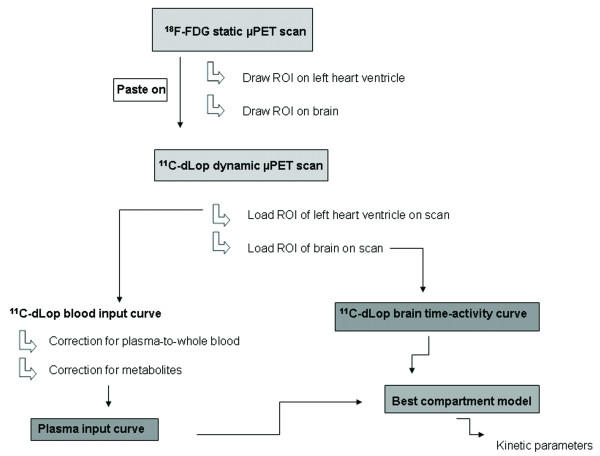
**An overview of the proposed method to determine a kinetic model of ^11^C-desmethylloperamide in mice**. A ^18^F-FDG static μPET scan is used to obtain the input function and the brain time-activity curve by drawing an ROI around the left heart ventricle and the brain.

Dynamic ^11^C-dLop PET data were sorted into frame sequences as mentioned above. The arterial blood input curve obtained from the μPET was corrected for plasma-whole blood ratio and metabolites. An ROI was signed around the whole brain on the ^18^F-FDG scan images and was used to determine the ^11^C-dLop brain time-activity curve (Figure 3). All data were loaded and analyzed with the PMOD software package (version 3.1., PMOD Technologies Ltd., Zurich, Switzerland).

Standardized uptake values (SUVs) were calculated using the following equation: *A*/(ID/BW), where *A *is the decay-corrected radioactivity concentration in the brain (measured in kilobecquerels per cubic centimeter), ID is the injected dose of ^11^C-dLop (measured in kilobecquerels), and BW is the mice body weight (measured in grams), resulting in SUVs expressed as grams per milliliter. To account for mice differences in the blood concentrations, which are the driving force for the brain concentrations, the brain-to-blood ratio was calculated using the SUVs in the blood and in the brain. A one-tissue compartment model was investigated, in which the rate constants *K*_1 _and *k*_2 _represent, respectively, the rate of transport from plasma to brain and the rate of outflow from the brain to the plasma. A two-tissue compartment model (with or without k4 fixed to 0) was also considered, since interaction of ^11^C-dLop in the brain might occur. The volume of vasculature was set as a variable in the compartment model.

### Statistical analysis

All calculated outcome parameters, differences between WT mice with and without cyclosporine, and KO mice were investigated with ANOVA and Bonferroni post hoc testing. The level of statistical significance was set to 5%.

## Results

### Radiosynthesis

Based on ^11^CH_3_I, ^11^C-dLop was prepared with a radiochemical yield of 32% (decay-corrected) and with a radiochemical purity of >95%. The specific activity averaged around 70 ± 2 GBq/μmol.

### Comparison of ^11^C-dLop left heart ventricle time-activity curve and blood counter measurement time-activity curve

**Figure 4:**
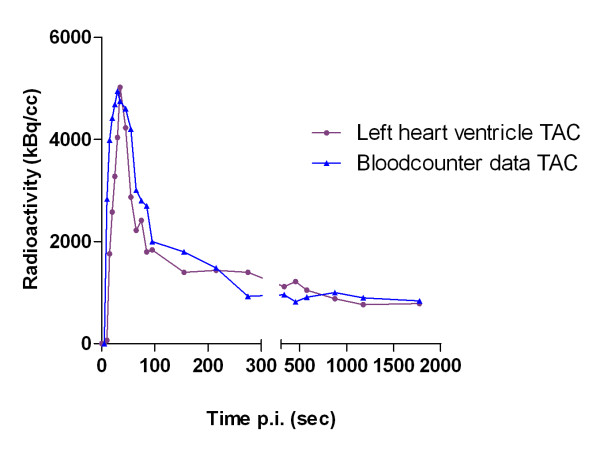
**Comparison of standard and new ^11^C-desmethylloperamide TAC**. Comparison of ^11^C-desmethylloperamide left heart ventricle time-activity curve (TAC) and blood counter dispersion corrected time-activity curve in a mouse.

The data from the blood counter were corrected for dispersion with *τ*_disp _calculated as 28 s. A comparison between the left heart ventricle time-activity curves and blood counter dispersion corrected time-activity curves showed acceptable agreement by graphical inspection (Figure 4). The AUC difference was 3.5% ± 4.2%, and the error percentage of the *K*_1_/*k*_2 _ratio was 6.5% ± 3.2%.

### Kinetic model for ^11^C-dLop

#### Determination of percent parent compound in plasma and plasma-whole blood ratio of ^11^C-dLop

The percent parent compound ^11^C-dLop at different time points p.i. in mice are summarized in Table 1. Statistical differences were observed either between WT and KO (*P *< 0.001) and between saline and cyclosporine pretreated WT mice (*P *< 0.001).

**Table 1:** Percentage of the parent compound (^11^C-dLop) in plasma

Mouse strain and pretreatment	**% **^**11**^**C-desmethylloperamide in plasma**		
	**1 min p.i**.	**10 min p.i**.	**30 min p.i**.
WT, saline	95 ± 1	72 ± 5	53 ± 3
WT, 50 mg cyclosporine/kg	95 ± 1	54 ± 5	23 ± 9
KO	98 ± 1	51 ± 1	34 ± 9

Percentage of the parent compound (^11^C-dLop) in plasma at 1, 10, and 30 min p.i. in different mouse strains and after different pretreatments. Results are expressed as percent of total radioactivity ± standard deviation. WT, wild-type mice; KO, knock-out mice.

Within the first half minute after ^11^C-dLop injection, the average ratio of tracer (^11^C-dLop and ^11^C-metabolites) plasma concentration to tracer (^11^C-dLop and ^11^C-metabolites) whole blood concentration was 0.67 ± 0.04. At 1 min after the tracer injection, the value dropped slightly to 0.49 ± 0.06, while at 3 min the ratio was restabilized to 0.67 ± 0.07. A mean ratio for all time points (0.64 ± 0.09) was further used as correction factor between blood and plasma.

#### PET data analysis and kinetic modeling of ^11^C-dLop

**Figure 5:**
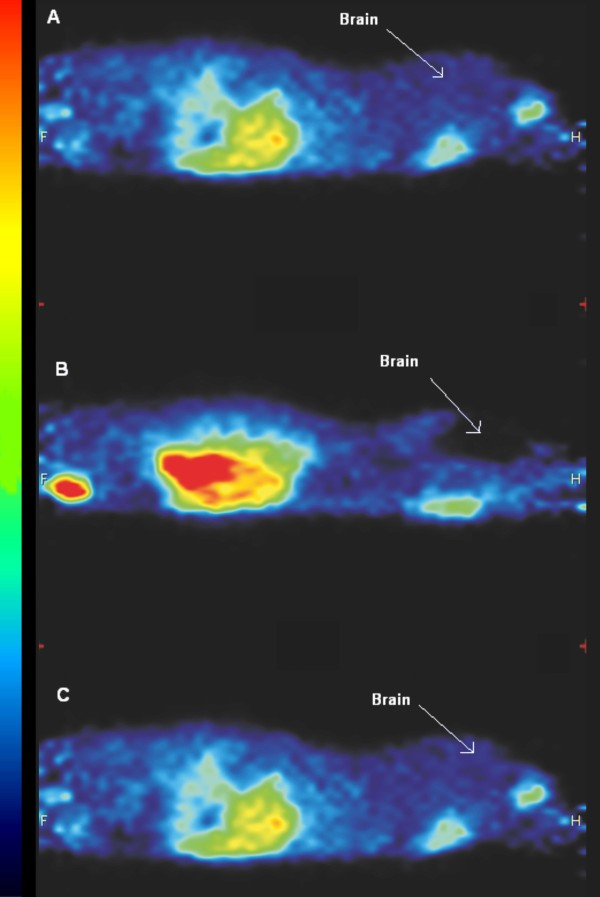
**Sagittal images of mice after intravenous administration of ^11^C-dLop**. Sagittal images of knock-out mice **(A)**, wild-type mice without cyclosporine pretreatment **(B) **and wild-type mice with cyclosporine pretreatment (50 mg/kg body weight, 30 min before tracer injection) (C), after intravenous administration of 20.0 ± 2.0 MBq of ^11^C-dLop. In each mice, the brains are indicated; the difference in tracer brain uptake between wild-type (no pretreatment), knock-out, and with cyclosporine-pretreated wild-type mice is clearly visible (color scale: black, lowest radioactivity uptake; red, highest radioactivity uptake).

**Figure 6:**
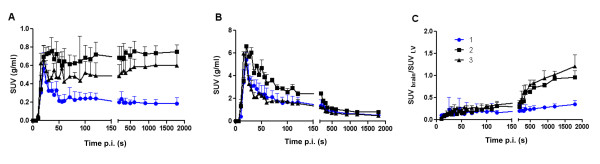
**Standard uptake values of ^11^C-desmethylloperamide**. SUVs of ^11^C-desmethylloperamide in wild-type mice with saline (1) or 50 mg/kg cyclosporine (2) pretreatment and in knock-out mice (3), expressed in grams per milliliter in function of time in brain **(A) **and in the left heart ventricle (LV) **(B)**. The ratio of SUV_brain_/SUV_LV _is depicted in graph **(C)**.

Differences in brain uptake of ^11^C-dLop were clearly observed (Figure 5). The brain SUVs calculated for KO, WT, and WT mice pretreated with cyclosporine were displayed in Figure 6A. In wild-type mice without pretreatment of cyclosporine, the average brain SUVs were 0.250, while in pretreated and KO mice SUVs were significantly higher (0.693 and 0.526, respectively). Although cyclosporine pretreatment of wild-type mice showed higher SUVs in the brain compared to knock-out mice, no statistical difference was observed (*P *> 0.05), probably due to larger standard deviations in knock-out mice. To exclude variation for the blood concentration over time between the different mice strains, SUVs were determined in the left heart ventricle. No statistical differences in the left heart ventricle SUVs (Figure 6B) were obtained. The brain-to-plasma SUVs are significant different between wild-type mice and KO mice and cyclosporine pretreated wild-type mice (Figure 6C).

**Figure 7:**
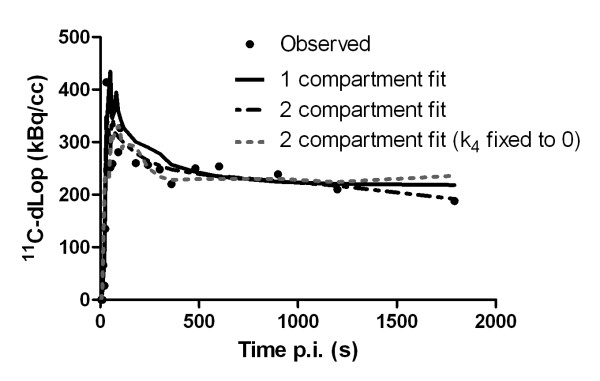
**One- and two-compartment model fittings for mice (*n *= 3), which were injected with ^11^C-dLop**. Circles represent observed μPET data taken from a region of interest drawn on the brain.

The two-tissue compartment model (with or without k4 fixed to 0) did not provide a significantly better fit than the one-tissue compartment model (Figure 7) (Akaike criterion values were in the same range). Moreover, the two-tissue compartment model estimated the kinetic parameters *K*_1 _and *k*_2 _with poorer identifiability than the one-tissue compartment model based on percent covariance values. Hence, Table 2 provides a summary of parameters estimated from the one-tissue compartment model with the noninvasive (left heart ventricle-based) method used to determine the input curve. *K*_1 _in WT mice is statistically smaller than *K*_1 _in knock-out mice (*P *= 0.008) and in cyclosporine-pretreated mice (*P *= 0.025), while the *k*_2 _is in the same range in all mice (*P *> 0.050). The differences between WT and knock-out mice or between saline and cyclosporine pretreatment in WT mice are also reflected in the *K*_1_/*k*_2 _ratio (*P *= 0.001).

**Table 2:** Summary of kinetic parameters

	***K***_**1 **_**(ml/cc/min)**	***k***_**2 **_**(1/min)**	***K***_**1**_**/*k***_**2**_
WT 1	0.054	0.190	0.28
WT 2	0.042	0.120	0.35
WT 3	0.027	0.059	0.46
KO 1	0.190	0.120	1.58
KO 2	0.230	0.095	2.42
KO 3	0.190	0.100	1.90
CYCLO 1	0.250	0.150	1.66
CYCLO 2	0.120	0.063	1.90
CYCLO 3	0.180	0.086	2.09

Summary of kinetic parameters estimated from the one-tissue compartment model for ^11^C-desmethylloperamide for all mice studied, using the noninvasive (left heart ventricle-based) method to determine the input curve. CYCLO, wild-type mice pretreated with 50 mg/kg cyclosporine, 30 min before tracer injection; KO, knock-out mice; WT, wild-type mice.

## Discussion

Biochemical process steps of a tracer in a tissue can be described by an appropriate tracer kinetic model. The behavior of a tracer is usually simplified and described by some mathematical kinetic compartments [11]. This model should be able to estimate the amount of radioactivity in each compartment, and the rate of exchange between these compartments. In PET imaging, these rate constants directly provide information on physiological parameters characterizing the behavior of the tracer in the tissue of interest. In case there is no reference region available, an arterial input curve is necessary to set up a kinetic model. Manual or automatic blood sampling is generally accepted as the gold standard to determine the arterial input curve. Nevertheless, in mice arterial sampling is technically difficult because of the relatively small diameters and fragility of the mouse blood arteries [12]. In addition, the total blood volume of a mouse is very limited (1.7 ml), making repeated blood sampling impossible without affecting the homeostasis of the mice [13]. Alternative methods to obtain an arterial input function are the use of a population database, based on a high number of mice or an arterial input function derived from PET images [14]. Attempts to determine the arterial input function in small animals from PET images were not convincing. Difficult delineation of the left heart ventricle on the PET scan in mice [15] or background signals from surrounding tissues in rats [16] were the main problems. Due to blurred ^11^C-dLop images on early as well as late time frames, it was impossible to delineate the left heart ventricle accurately. We therefore propose a new image-derived method, using a ^18^F-FDG scan after a ^11^C-dLop scan. Unlike ^11^C-dLop, ^18^F-FDG shows a selective uptake in the myocardium [17-19], making the determination of the left ventricle easy without the problem of spill-in of activity from the surrounding lungs. A comparison between the left heart ventricle time-activity curves (alternative method) and blood counter time-activity curves (corrected for dispersion) showed acceptable graphical agreement. A small AUC difference (3.5%) was observed compared to Green *et al. *(18%) [20], who did not use a ^18^F-FDG scan to delineate the left heart ventricle, but instead a small ROI based on the highest activity in the aorta area on the earliest time frames. Also, the comparison of the *K*_1_/*k*_2 _ratio showed analog correlations (6.5%) between standard blood sampling and our proposed method. However, one must realize that the usefulness of our method must be validated for each radioligand because determination of the arterial input function based on the left ventricle could lead to a poor resemblance with the blood sampling input curve especially for radioligands with high myocardial uptake.

Both in wild-type and in P-gp knock-out mice, the percent of parent compound was investigated, resulting in variations probably due to the influence of cyclosporine or to an adaptation of the body to the absence of P-gp efflux transporters. These differences are not an obstacle concerning our experiment because the latter correction was introduced to take these differences into consideration.

Variations in ^11^C-dLop brain uptake between wild-type and knock-out/cyclosporine-pretreated mice were clearly observed in μPET images and SUVs. Moreover, differences in ^11^C-dLop uptake in the intestines were observed and could be explained by the absence of P-gp in KO mice resulting in a lower tracer uptake, while in WT mice P-gp located in the intestines pumps the tracer out of the blood into the intestines, resulting in higher uptake. The higher radioactivity in the abdomen of WT mice, as observed in Figure 5, could also be explained as higher uptake in the liver, which is in accordance with results obtained in humans [21]. Nevertheless, kinetic parameters obtained from a compartment model will provide useful mathematical information about the behavior of the tracer. Since no statistical difference in model fittings between the one- and two-compartment model was observed, the simplest model, meaning the one-tissue compartment model, was preferred. This is in accordance to the results mentioned by Kreisl *et al. *[22]. In a one-tissue compartment model, the tracer behaves in a straightforward manner explained by an uptake in the brain with a speed, represented by the kinetic parameter *K*_1_, and efflux out of the brain described by *k*_2_. *Binding *with any receptors in the brain or metabolisation of the tracer in the brain will not occur in this model. Lazarova *et al. *[6] already mentioned that ^11^C-dLop showed no clinical relevant interaction with the opiate receptors in the brain.

The kinetic parameters *K*_1 _and *k*_2 _obtained from a one-tissue compartment model of ^11^C-dLop were evaluated in WT, KO, and WT mice pretreated with cyclosporine. One should expect that *K*_1_, which represents the passive influx of the tracer in the brain, should not change between the different groups. *k*_2_, which represents the efflux out of the brain by P-gp transport, was supposed to be lower in KO mice and in the WT mice pretreated with cyclosporine. Our data showed that the *K*_1 _was statistically lower in WT mice compared to KO or cyclosporine-pretreated WT mice, while the *k*_2 _was very similar in all tested mice. Kreisl *et al. *[22] reported the same result after blockage of the P-gp with tariquidar and suggested that tariquidar increased brain uptake of ^11^C-dLop by increasing its entry (*K*_1_) rather than by decreasing its efflux (*k*_2_). The substrate is captured in the endothelial cells, before it enters the intracellular compartment. Therefore, if P-gp captures all of the substrate while in transit through the membrane, its effect is entirely on *K*_1_. If some of the substrate escapes and has time to interact with the intracellular milieu, and if there is an efflux from the cell, P-gp will both decrease *K*_1 _and increase *k*_2 _[23]. Nevertheless, we think that also a time influence of the P-gp transport should be taken into consideration. The course of the brain SUV curve (Figure 6A) in WT mice demonstrates a fast uptake in the brain, followed by a rapid wash out of the brain, resulting in an SUV of 0.25 already 1 min after the tracer injection, while in KO and pretreated WT mice also a fast uptake was observed, followed by an accumulation in the brain of the tracer combined with a slow efflux. The observed different course of the brain curve between WT and KO mice, even as between cyclosporine pretreated WT mice suggests that the duration of the scan could play an important role on the determination of the kinetic parameters in the kinetic model.

**Figure 8:**
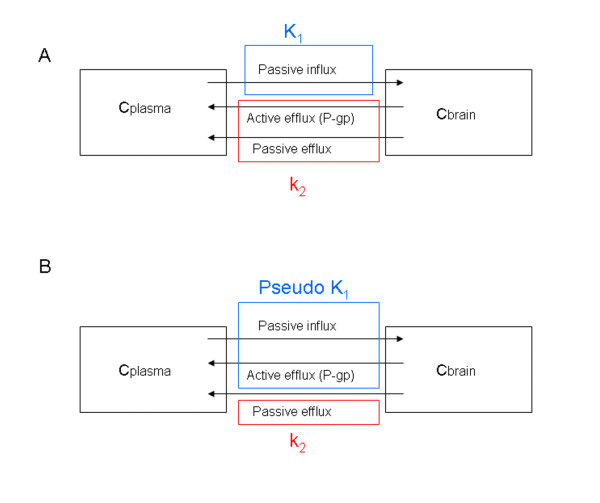
**Schematic representation of different methods to determine the rate constants obtained from one-tissue compartment model**. **(A) **Rate constants *K*_1 _and *k*_2_, obtained from one-tissue compartment model with all scan data incorporated. *K*_1 _represents the passive influx, while *k*_2 _is a combination of active and passive efflux. **(B) **shows rate constants obtained from a one-tissue compartment model with only the first 2 min of the scan data. Pseudo *K*_1 _is defined as a combination of the passive influx and active efflux, but *k*_2 _only represents passive efflux. C, concentration of ^11^C-dLop.

This hypothesis was substantiated by the results of *K*_1 _and *k*_2 _obtained in a one-tissue compartment model with incorporation of only the first 2 min of dynamic scanning. These results showed a statistically higher *k*_2 _in WT mice (8.0 ± 0.1) compared to KO (2.3 ± 0.9; *P *= 0.070) and compared to cyclosporine pretreated WT mice (1.5 ± 0.5; *P *= 0.002), while *K*_1 _was statistically not different between the different groups (*P *> 0.05). This means that during the first 2 min after administration of ^11^C-dLop, efflux out of the brain is dominated by efflux transporters, while at later time points passive diffusion is more important. The *K*_1_/*k*_2 _ratio of WT obtained with the 2-min scan data were statistically different compared to the ratios in KO and compared to cyclosporine-pretreated WT mice. So, we propose *K*_1 _as a pseudo value, representing a combination of passive influx of ^11^C-dLop through the BBB and a rapid energy dependent output by P-gp, while *k*_2 _corresponds to slow passive efflux out of the brain (Figure 8).

## Conclusion

The use of an easy to implement ^11^C-desmethylloperamide kinetic model in mice for imaging P-gp function is presented without arterial blood sampling. The method to determine the input function is based on the delineation of an ROI on the ^18^F-FDG scan images and using this ROI on images obtained from a dynamic scan with ^11^C-dLop. The *K*_1 _or *K*_1_/*k*_2 _ratio obtained from the ^11^C-dLop tracer kinetic model is a good parameter for the active P-gp rate and can be applied in future experiments to evaluate the role of the upregulation of P-gp in psychotropic drug resistance, such as refractory epilepsy and in tumor resistance to therapy.

## Abbreviations

AED: antiepileptic drugs; AUC: area under the curve; BBB: blood-brain barrier; BW: mice body weight; ^11^C-dLop: ^11^C-desmethylloperamide; DMF: dimethylformamide; DMSO: dimethylsulfoxide; ID: injected dose; i.v.: intravenously; KO: P-glycoprotein knock-out mice; P-gp: P-glycoprotein; p.i.: post injection; SUVs: standardized uptake values; TBAH: tetrabutylammoniumhydroxide; WT: wild-type mice.

## Competing interests

This work was supported and funded by a Ph.D. grant of the Institute for the Promotion of Innovation through Science and Technology in Flanders (IWT-Vlaanderen). Research work of Dieter De Naeyer was also funded by FWO-Vlaanderen. Prof. Paul Boon has received fees for presentations and travel grants from UCB Pharma and Janssen-Cilag. The remaining authors have no conflicts of interest.

## Authors' contributions

LM designed and carried out the experimental studies and has written the manuscript. DD has investigated and corrected the blood plasma curve for dispersion. PB and FD participated in the design of the study and helped to draft the manuscript. The manuscript has been seen and approved by all authors.
